# Comprehensive Identification of Meningococcal Genes and Small Noncoding RNAs Required for Host Cell Colonization

**DOI:** 10.1128/mBio.01173-16

**Published:** 2016-08-02

**Authors:** Elena Capel, Aldert L. Zomer, Thomas Nussbaumer, Christine Bole, Brigitte Izac, Eric Frapy, Julie Meyer, Haniaa Bouzinba-Ségard, Emmanuelle Bille, Anne Jamet, Anne Cavau, Franck Letourneur, Sandrine Bourdoulous, Thomas Rattei, Xavier Nassif, Mathieu Coureuil

**Affiliations:** a Institut Necker Enfants-Malades, INSERM U1151, Equipe 11, Paris, France; b Université Paris Descartes, Sorbonne Paris Cité, Faculté de Médecine, Paris, France; c Department of Infectious Diseases and Immunology, Faculty of Veterinary Medicine, Utrecht University, Utrecht, The Netherlands; d CUBE Division of Computational Systems Biology, Department of Microbiology and Ecosystem Science, University of Vienna, Vienna, Austria; e Plateforme Génomique de l’Institut Imagine, Hôpital Necker, Paris, France; f INSERM U1016, Institut Cochin, CNRS UMR8104, Paris, France; g Unidade de Microbiologia Molecular e Infecção, Instituto de Medicina Molecular, Lisbon, Portugal; h Assistance Publique—Hôpitaux de Paris, Hôpital Necker Enfants Malades, Paris, France

## Abstract

*Neisseria meningitidis* is a leading cause of bacterial meningitis and septicemia, affecting infants and adults worldwide. *N. meningitidis* is also a common inhabitant of the human nasopharynx and, as such, is highly adapted to its niche. During bacteremia, *N. meningitidis* gains access to the blood compartment, where it adheres to endothelial cells of blood vessels and causes dramatic vascular damage. Colonization of the nasopharyngeal niche and communication with the different human cell types is a major issue of the *N. meningitidis* life cycle that is poorly understood. Here, highly saturated random transposon insertion libraries of *N. meningitidis* were engineered, and the fitness of mutations during routine growth and that of colonization of endothelial and epithelial cells in a flow device were assessed in a transposon insertion site sequencing (Tn-seq) analysis. This allowed the identification of genes essential for bacterial growth and genes specifically required for host cell colonization. In addition, after having identified the small noncoding RNAs (sRNAs) located in intergenic regions, the phenotypes associated with mutations in those sRNAs were defined. A total of 383 genes and 8 intergenic regions containing sRNA candidates were identified to be essential for growth, while 288 genes and 33 intergenic regions containing sRNA candidates were found to be specifically required for host cell colonization.

## IMPORTANCE

Meningococcal meningitis is a common cause of meningitis in infants and adults. *Neisseria meningitidis* (meningococcus) is also a commensal bacterium of the nasopharynx and is carried by 3 to 30% of healthy humans. Under some unknown circumstances, *N. meningitidis* is able to invade the bloodstream and cause either meningitis or a fatal septicemia known as purpura fulminans. The onset of symptoms is sudden, and death can follow within hours. Although many meningococcal virulence factors have been identified, the mechanisms that allow the bacterium to switch from the commensal to pathogen state remain unknown. Therefore, we used a Tn-seq strategy coupled to high-throughput DNA sequencing technologies to find genes for proteins used by *N. meningitidis* to specifically colonize epithelial cells and primary brain endothelial cells. We identified 383 genes and 8 intergenic regions containing sRNAs essential for growth and 288 genes and 33 intergenic regions containing sRNAs required specifically for host cell colonization.

## INTRODUCTION

*Neisseria meningitidis* (meningococcus) is a common inhabitant of the human nasopharynx, and as such it is a normal saprophytic organism that is transmitted from person to person by direct contact and/or aerosol transmission. *N. meningitidis* is also responsible for meningitis and for a thrombotic/leakage syndrome that, in its severe form, causes an extensive necrotic purpura with massive vascular leakage and shock, a condition known as purpura fulminans (1).

*N. meningitidis* is highly adapted to nasopharyngeal colonization and is capable of regulating multiple pathways involved in iron acquisition, adhesion, and metabolism (2–4). This adaptation is directly linked to the physical properties of the nasopharyngeal niche, like temperature (5) and oxygen concentration (6). Although many meningococcal virulence factors have been identified, the mechanisms that allow the bacterium to switch from the commensal to pathogen state remain unknown. One of the important peculiarities of meningococcal pathogenesis is the very uncommon interactions of bacteria with the human mucosa and the peripheral microvasculature, especially endothelial cells lining the blood-brain barrier; these interactions lead to major vascular dysfunction and bacterial entry into the brain, respectively (7, 8). Adhesion to human cells requires mainly type IV pili, which are long retractable filaments also involved in aggregation and competence, while the secondary adhesin-like Opa and Opc proteins may be specifically involved in adhesion to specific cell types (9). However, colonization and communication with the different human cell types, which are major aspects of the *N. meningitidis* life cycle, are still poorly understood.

The availability of high-throughput DNA sequencing technologies has emerged as the de facto means to detect variations in genetic fitness of individual members of a very large pool of mutants undergoing selection in infected hosts. Transposon (Tn) insertion site sequencing, also known as Tn-seq, is a powerful analytical method that allows the comparative contribution of bacterial genes in its host (10–12). By comparing quantitative levels in different populations of mutated genes that contribute to specific phenotypes, Tn-seq gives unique insights into the role of individual genes and their regulators.

Here, we engineered highly saturated random transposon insertion libraries of *N. meningitidis*. Using the high-throughput insertion tracking by deep sequencing (HITS) strategy (11), we assessed the fitness of mutations within the libraries during routine growth and that of colonization of endothelial or epithelial cells in a microfluidic flow device to mimic physiologic micromechanical environments subjected to fluid flow (13). We found that 18% of the *N. meningitidis* open reading frames (ORFs) and 8 intergenic regions (IRs) containing sRNA candidates are essential for routine growth, while 19% of all ORFs and 66 IRs containing sRNA candidates are directly involved in cell colonization.

## RESULTS

**FIG 1:**
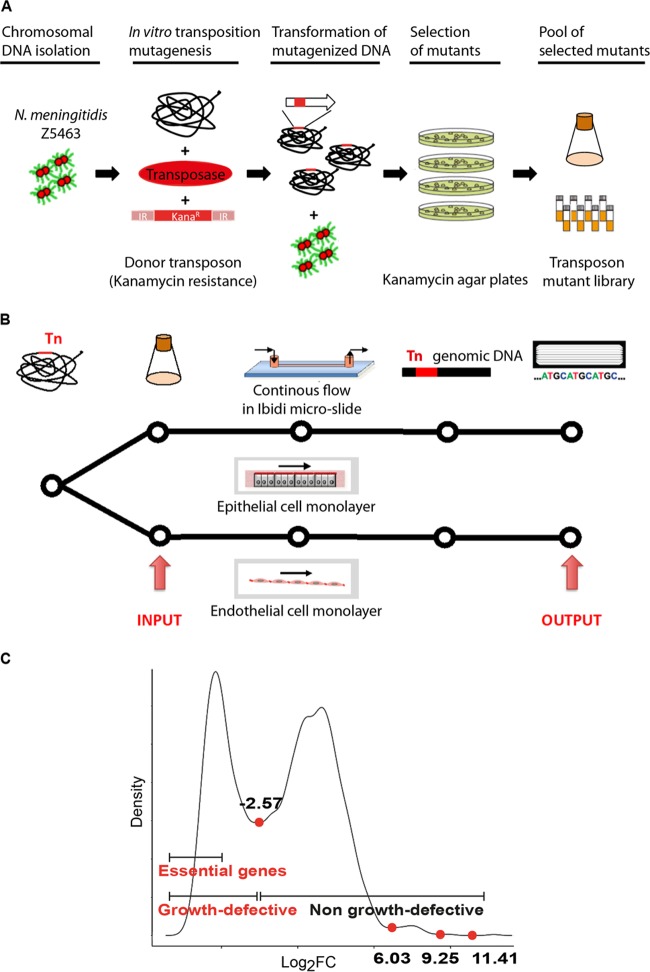
Schematic representation of the Tn-seq screen for identification of genes involved in epithelial and endothelial cell colonization. (A) *N. meningitidis* mutant libraries were constructed via random insertion of a transposon carrying a kanamycin resistance cassette. Mutants were pooled from a 2-h outgrowth culture and stored at −80°C. (B) Aliquots of the Tn mutants bank cultures were thawed and grown until mid-log phase (the input pools); input pools were used to infect an epithelial or endothelial cell monolayer in parallel on a microslide (ibidi, Germany) and recovered 18 h later (output pools). The presence of each mutant was investigated in both input and output pools by Tn-seq analysis. (C) Density plot of log_2_ fold changes of measured read counts versus expected read counts. The log_2_-transformed ratio of measured over expected read counts of insertion sites was used to generate a kernel density plot with a Gaussian model with stepwise increasing bandwidth and 2,048 bins, until a single local minimum was found between the two major distributions. The local minimum was detected by calculating the first derivative of the density and by locating the position where it traversed from values below zero to values above zero. This fold change value corresponds to a value closest to the minimum between the peaks of essential and nonessential genes and was used as a cutoff to determine whether a mutant was growth defective or not. A second cutoff of −5 was chosen arbitrarily as a cutoff for gene essentiality, as this value represents the left-most part of the results with growth-defective mutants. At this cutoff, the possibility of a false positive is negligible.

The aim of our study was to identify the core essential genome of *N. meningitidis* and to identify genes required for endothelial and/or epithelial cell colonization. We used the flow colonization model described by Jamet et al. (13) combined with a Tn-seq approach. To answer these questions, we generated three independent transposon mutant libraries by random insertion of a low-insertion-specificity transposon (Fig. 1A). Bacteria were allowed to adhere to endothelial or epithelial cell monolayers under flow conditions to mimic the physiologic micromechanical environments (Fig. 1B).

### Identification of core essential genes.

In order to interpret the data obtained for bacterial colonization of host cells, we first had to identify the core essential genes. To perform this task, cultures of mutant isolates from each of the 3 libraries were independently grown for 2 h in epithelial or endothelial cell culture medium (CCM) (Table 1), and genomic DNA was extracted from each library and sequenced following an adapted HITS approach, as described in Materials and Methods and in reference 11. We analyzed the input libraries, designated InEpi.1 to InEpi.3 and InEndo.1 to InEndo.3, by using the ESSENTIALS tool kit (14). We first confirmed that input libraries grown in endothelial or epithelial CCM were not statistically different, based on the log_2_ fold change (log_2_ FC) of each gene’s expression level (data not shown).

**TABLE 1:** Tn sequencing, processing, and mapping results

Cell type, library, and group	No. of reads			
	Sequenced	Processed	Mapped	TIS flanks^a^
Epithelial cells				
Library 1				
InEpi.1A	18,169,002	1,967,796	1,854,352	91,729
OutEpi.1A	20,311,714	2,031,700	1,957,901	35,848
Library 1, second analysis				
InEpi.1B	7,612,788	523,918	499,704	43,514
OutEpi.1B	7,643,004	674,595	656,482	16,130
Library 2				
InEpi.2	10,222,928	795,475	758,817	52,608
OutEpi.2	8,938,418	772,379	752,042	21,346
Library 3				
InEpi.3	8,602,318	681,983	649,809	56,853
OutEpi.3	6,679,952	615,498	599,600	18,656
Endothelial cells				
Library 1				
InEndo.1	8,577,338	708,680	677,257	54,444
OutEndo.1	8,116,488	716,420	693,786	30,648
Library 2				
InEndo.2	8,185,084	705,901	673,945	56,959
OutEndo.2	7,152,626	636,686	616,375	31,444
Library 3				
InEndo.3	8,115,038	647,848	618,049	57,081
OutEndo.3	10,303,180	851,806	822,415	37,669

a TIS, transposon insertion sites.

**FIG 2:**
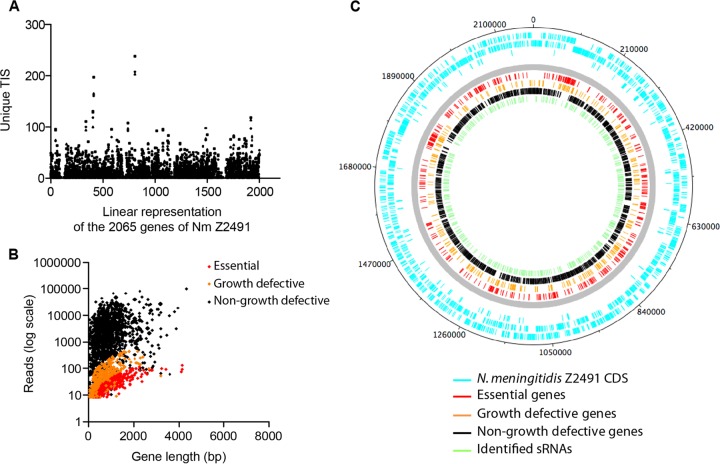
Characterization of the transposon mutant libraries. (A) Distribution of unique TIS among the 3 input libraries along the sequenced genome of *N. meningitidis* Z2491. (B) The number of transposon-containing reads per ORF in the input pool, in relation to gene length, is shown for essential genes, growth-defective genes, and non-growth-defective genes. Essentiality was determined based on the ratio of the number of reads per gene divided by the expected number of reads per gene, which depends on the size of the gene and the number of transposon insertion sites. Thus, a large gene with few transposon insertion sites may not be essential, even though few reads are mapped within that gene. (C) Distribution of the CDS containing random Tn insertions on the genome map of *N. meningitidis* Z2491 and of the sRNAs identified by RNA-seq. In the outer circles, bars indicate *N. meningitidis* Z2491 CDS. In the inner circles, bars indicate the essential genes, the nonessential but growth-defective genes, and the non-growth-defective genes in routine cell culture medium (according to the color key shown in the figure); the bars on the innermost circle indicate the location of the 390 identified sRNAs.

The total number of unique transposon insertion sites (TIS) for the 6 libraries combined was 38,566, which corresponds to an average distance between transposon insertions of 57 bp. This number of TIS corresponds to a saturation of 99.99% of the 2.18 Mbp of the Z5463 chromosome, according to Poisson’s law (see Table S1B in the supplemental material) (15). A rarefaction analysis confirmed the saturation of the 3 different input libraries (see Table S1B). The location of the insertions showed an even distribution around the chromosome (Fig. 2A).

Gene essentiality of Tn-seq data sets was predicted by using the ESSENTIALS tool set (14), which enabled us to calculate a statistical essentiality metric for each ORF. The log_2_ FC was assigned to each ORF based on a comparison of the expected number of reads versus the measured number of reads (see Materials and Methods for more details). The results corresponding to each gene are reported in Table S1A in the supplemental material. A density plot of the log_2_ FC was generated (Fig. 1C) and indicated that all genes with a log_2_ FC value of less than −2.57 were statistically significantly impaired for growth. Within the growth-defective population, we arbitrarily considered essential the ORFs with a log_2_ FC value less than −5. Three gene populations were thus categorized: the essential genes (log_2_ FC less than −5), the nonessential genes causing a growth defect (log_2_ FC between −2.57 and −5), and the genes that did not cause a growth defect (non-growth-defective genes; log_2_ FC greater than −2.57). Essential, growth-defective, and non-growth-defective genes formed well-resolved populations when plotted as a function of the number of reads per gene per gene length (Fig. 2B).

Considering that expression of a gene is mandatory for defining its essentiality, we removed from our analysis untranscribed genes, based on the high-throughput RNA sequencing (RNA-seq) analysis as described in Materials and Methods. A genomic region was considered transcribed if it had an RPKM (reads per kilobase of gene per million mapped reads) value greater than 2.3. Out of a total of 1,994 coding DNA sequences (CDS; not including the 12 rRNAs and 58 tRNAs), 1,831 were above the transcriptional threshold (92%), whereas 163 CDS were below (8%) and therefore not expressed under these experimental conditions and were removed from the analysis. Most of the untranscribed genes corresponded to hypothetical proteins. Among the genes that were not found to be transcribed, we did not find important genes for meningococcal virulence, except for *hpuA*, the hemoglobin-haptoglobin utilization lipoprotein A that is involved in iron acquisition. In addition, 44 ORFs for which no reads could be assigned in the Tn-seq analysis were also excluded from this analysis (see Table S1A in the supplemental material). Fifteen of these 44 genes encoded 9 rRNA sequences (5S, 16S, or 23S) and 6 tRNA sequences. Some of these 44 ORFs are likely to be core essential ORFs; however, we cannot exclude that some of these 44 genes represented false-negative results.

This analysis resulted in the identification of 383 transcribed genes of strain Z5463 that were essential for growth in CCM, representing 19% of the genome (see Table S1A in the supplemental material). This is consistent with earlier studies, which reported that 15% to 25% of all genes of a bacterial chromosome are essential (16–18). Besides, 329 genes were classified as nonessential but growth defective, representing 16% of the genome (see Tables S1A and S3). Essential, growth-defective, and nonessential genes showed an even distribution around the chromosome (Fig. 2C).

### Identification of intergenic regions containing sRNAs essential for growth of *N. meningitidis.*

Many transposon insertion sites were contained within IRs. These regions contained promoter sequences, regulatory elements, and putative sRNA sequences. To identify essential intergenic regions that possibly contain sRNA, we first identified noncoding sRNAs expressed in intergenic regions by using RNA sequencing of wild-type strain Z5463. As described in the methods section of Text S1 in the supplemental material, cDNA libraries were constructed and sequenced by using Ion Torrent technology (see Table S2 in the supplemental material). Both rRNAs and tRNAs were excluded from the analysis. A total of 390 sRNAs were identified in IRs, and these were distributed all along the meningococcal genome (Fig. 1C; see also Table S2). Out of these, 30 were located in IRs already described to contain sRNAs by Fagnocchi and coworkers (19) (see Table S2G).

We further analyzed the essentiality of the IRs containing these 390 sRNAs as we had previously analyzed the essential genes with Tn-seq (see Table S1E in the supplemental material). Considering the size of the IR, and according to Poisson’s law, 93 of the 390 IRs containing sRNA candidates had a probability higher than 5% of not having a transposon insertion because of their small size. These 93 IRs were therefore excluded from the analysis. By comparing the expected number of reads to the obtained number of reads within these IRs, we found that 8 IRs that contained sRNA candidates were essential for growth (log_2_ FC < −5), and 47 were growth defective (−5 < log_2_ FC < −2.57) (see Table S2B in the supplemental material). Besides, the presence of these sRNA candidates in a flanking gene promoter was verified with the PromBase tool. Only 3 among these 55 sRNAs were located in a promoter region associated with an essential or growth-defective gene (see Table S2). These 3 sRNAs belonged to the group of 47 sRNAs which, when mutated, were responsible for growth-defective strains. Accordingly, we could not exclude the possibility that the phenotype associated with a mutation in these 3 sRNAs was due to an effect on the downstream genes.

### Analysis of the core essential genes.

**FIG 3:**
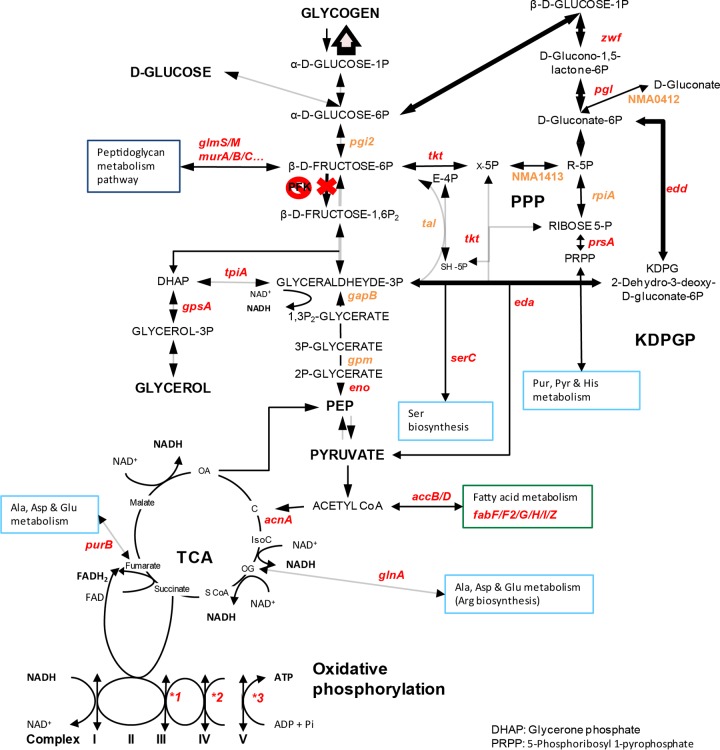
Schematic representation of the main metabolic pathways of essential genes for routine growth of *N. meningitidis*. The main essential genes for growth on GCB agar plates and CCM are highlighted in red, and the genes that encode proteins that cause a severe growth defect are highlighted in orange. *1, essential genes *petA*, *-B*, and *-C*; *2, essential genes *ccoO* and *-N*; *3, essential genes *atpA*, *-B*, *-D*, *-F*, and *-G* and growth-defective genes *atpE* and *-H*.

Of the 383 genes found to be essential for growth in *N. meningitidis*, 33% were involved in metabolism, 27% in information storage and processing, and 17% in cellular processes and signaling (see Table S3 in the supplemental material). Essential genes implicated in information storage and processing were mainly translation factors (*rplA*, -*B*, -*C*, -*D*, -*E*, -*F*, -*M*, -*P*, -*Q*, -*R*, -*S*, -*U*, -*V*, -*X*, and -*Y*) and transcription factors (*rpoA*, *-B*, -*C*, -*D*, and -*H* and *nusA*). Essential genes involved in metabolism are depicted in Fig. S2A in the supplemental material. Among them, genes of the pentose phosphate pathway (PP pathway) and of the Entner-Doudoroff pathway (2-keto-3-deoxy-6-phosphogluconate pathway [KDPGP]), the two alternative routes to glycolysis, were selected (Fig. 3). The genes *tkt* and *prsA* for PP pathway enzymes, which lead to the production of PRPP (phosphoribosylpyrophosphate) involved in the *de novo* synthesis of purines, pyrimidines, histidine, tryptophan, and pyridine nucleotides, were essential, together with the 4 main enzymes of the KDPGP, *zwf*, *pgl*, *edd*, and *eda*. Moreover, 5 other enzymes of the PP pathway (NMA0412, NMA1413, *rpiA*, *tal*, and *pgi2*) were growth defective. The use of these alternative routes to catabolize glucose is consistent with the fact that *N. meningitidis* lacks the phosphofructokinase enzyme, which plays a central role in glycolysis (20, 21). Interestingly, production of NADPH in the PP pathway was also described as critical for virulence of *Salmonella enterica* serovar Typhimurium, another Gram-negative bacterium (22). Besides, *gapB* (NMA0246), one of the two glyceraldehyde-3-phosphate dehydrogenases (GAPDHs) involved in the second part of glycolysis (production of pyruvate from glyceraldehyde-3P), had a growth defect, and 2 genes involved in the gluconeogenesis pathway starting from glycerol (*gpsA*, *tpiA*) were also essential. As expected, essential genes were also involved in three of the five oxidative phosphorylation complexes (III, IV, and V; which is the ATP synthase) and that of the ubiquinone biosynthesis pathway (*ubiA*, *ubiE*, and *ubiG*). Furthermore, two enzymes involved in the denitrification pathway (reduction of nitrite to nitrous oxide via nitric oxide), AniA and NorB, were found to be essential (see Table S4 in the supplemental material). This pathway, described by Rock et al., is an alternative to respiration for *N. meningitidis* when oxygen is restricted (23).

Our screen also identified essential genes implicated in fatty acid metabolism and in alanine, aspartate, and glutamate metabolism (Fig. 3; see also Fig. S1A and Table S3 in the supplemental material). The essential genes involved in the two latter pathways (*glnA*, *purB*, *fabF*, *-F2*, -*G*, -*H*, -*I*, and -*Z*, and *accB* and *-D*) are linked to the tricarboxylic acid (TCA) cycle (Fig. 3). Production of coenzyme A through the proteins encoded by the *panC*, *birA*, and *coaD* genes is also critical. Besides, a link between fructose-6P, a substrate of the sugar catabolic pathway and the peptidoglycan metabolism pathway, was found to be essential (genes *glmS* and *-N*, NMA0284, *murA*, *-B*, *-C*, *-D*, *-E*, and *-G*, NMA2068, *mraY*, NMA2072, NMA0665, and NMA1095).

Many genes involved in biosynthesis of secondary metabolites are essential for growth. Genes involved in vitamin B_6_ biosynthesis, *pdxH* and -*J* and *serC* are categorized as essential and *pdxA* as growth defective. NMA1262, NMA0958, NMA1950, NMA0896, and NMA2179, which are involved in folate biosynthesis, appear to be essential, while *thiD*, *-G*, and *-L*, NMA0363, and NMA0364, which are involved in thiamine biosynthesis, have log_2_ FC values between −2.57 and −5.

Meningococcal lipooligosaccharide plays a crucial role in bacterial host survival due to its ability to resist human serum (24, 25). Indeed, genes involved in lipooligosacharide metabolism, such as *lpxB*, *-D*, *-H*, *-K*, and *-L*, have a log_2_ FC value less than −5, while insertion of transposons within sequences of *lpxA* and *-C*, *kdtA* and -*S*, NMA2134, and NMA2135 is clearly detrimental for growth (−5 < log_2_ FC < −2.57).

Additionally, we found essential genes related to iron metabolism (reported in Table S4 in the supplemental material), including the ferric uptake transcriptional regulator *fur*. Among the essential transporter proteins, we found 11 members of the ATP-binding cassette (ABC) family, 5 of which are transporters involved in manganese transport (NMA0790 and NMA0791), magnesium transport (*mgtE*), phosphate transport (*pit*), or potassium transport (*trk*). We also annotated 7 ABC transporters to be growth-defective transporters.

Bacteria have developed two-component systems (TCSs) in order to sense and respond to changes in many different environmental conditions (26). Among the four TCS genes encoded in the meningococcus genome (20), we found the NMA0797 PhoQ (MisS)-NMA0798 PhoP (MisR) system and the putative two-component system transcriptional regulator protein NMA0159 to be essential for growth on cell culture media (see Table S4 in the supplemental material). Interestingly, an enzyme involved in proper folding of periplasmic, secreted, and membrane proteins, DsbD, which is regulated by the MisS-MisR TCS, was also found to be essential (27).

Recent studies have demonstrated that *mafB* genes encode polymorphic toxins that provide an advantage in competition assays (28, 29). In meningococcal strains, *mafB* genes are present on three Maf genomic islands, termed MGI-1, -2, and -3. Immediately downstream of each *mafB* gene, a *mafI* gene encodes a specific immunity protein which protects the bacterium against self-intoxication and against toxins from neighboring bacteria (28). As expected, we found that the three immunity genes associated with the three *mafB* genes are either essential for growth of *N. meningitidis* (NMA0323) or results in growth defects when mutated (NMA2114 and NMA0854) (see Table S4 in the supplemental material). Interestingly, several immunity genes (NMA2116, NMA2117, and NMA2118) and a cassette encoding an alternative toxic C terminus (NMA2115) in MGI-1 are essential for growth.

### Selection of genes required for colonization of human cells.

**FIG 4:**
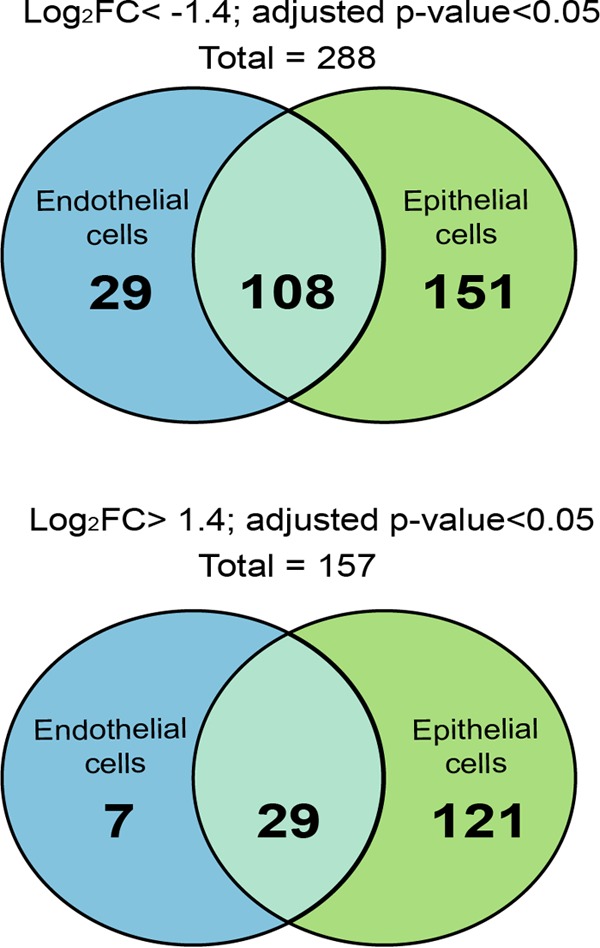
Identification of conditional essential genes necessary for colonization of epithelial and endothelial cells. The Venn diagram shows the absolute number of necessary ORFs (log_2_ FC < −1.4; adjusted *P* < 0.05) and beneficial ORFS (log_2_ FC > 1.4; adjusted *P* value < 0.05). (ORFs were selected in both the epithelial cell and endothelial cell *in vitro* models.)

The input libraries were used to infect VI cells on microslides (ibidi, Germany) containing monolayers of Fadu nasopharyngeal epithelial cells or hCMEC/D3 brain microvessel endothelial cells (Fig. 1B). A continuous flow of CCM (flow rate, 0.04 ml/min) was applied for 18 h to the cell monolayer, starting 1.5 h after infection. This flow rate was chosen to obtain a permanent renewal of the CCM to allow efficient colonization of the cells. Bacteria of these output libraries were then harvested. These output libraries were designated OutEpi.1 through -3 and OutEndo.1 through -3 (Fig. 1B). En masse sequencing of the input and output pools allowed us to calculate the fitness of genes for colonization of cells *in vitro*. Using the ESSENTIAL tool kit, a log_2_ FC of each gene was obtained from three independent experiments (see Table S1D in the supplemental material) (see Materials and Methods for details regarding log_2_ FC calculations). We arbitrarily considered that a gene having a log_2_ FC less than −1.4 and an adjusted *P* value of <0.05 was necessary for host cell colonization, while a gene with a log_2_ FC greater than 1.4 and an adjusted *P* value of <0.05 on the other hand favored host cell colonization. Comparison of the data sets revealed a total of 288 genes important for colonization, from which 108 were common to both human cell types and 151 and 29 genes were specifically selected during colonization of epithelial or endothelial cells, respectively (Fig. 4; see also Table S3 in the supplemental material). On the other hand, a total of 157 ORFs were found to be beneficial for this phenotype. Twenty-nine ORFs were common to both human cell types, and 121 and 7 were specifically selected after passage on epithelial and endothelial cells, respectively (Fig. 4; see also Table S3), thus indicating that colonization of epithelial and endothelial cells has mutual and distinct requirements. In addition, these data suggested that epithelial cell colonization is likely to be more demanding for the bacteria than is endothelial cell colonization.

### Analysis of the genes important for colonization onto both epithelial and endothelial cells. (i) Glucose metabolism (7 genes).

The 108 genes whose transposon-induced disruption significantly lowered fitness of both epithelial and endothelial cells included genes important for bacterial metabolism, such as carbohydrate or amino acid metabolism or in complex I of the oxidative phosphorylation system. Remarkably, several genes implicated in glycolysis or gluconeogenesis were negatively selected (*fbp*, *fba*, *gapA*, *pgk*, and *pykA*). The importance of *fbp* expression, which is specific to the gluconeogenesis pathway, suggests that the production of β-d-fructose-6P is crucial for cell colonization. Interestingly, *gapA* has been demonstrated to play an important role in adhesion to both human epithelial and endothelial cells (30). Besides, accumulation of phosphoenolpyruvate (PEP) may be crucial for colonization, since two genes leading to PEP (*pykA* and *ppc*) had reduced fitness and two genes involved in the PEP-to-citrate pathway (*lpdA2* and *gltA*) had a beneficial effect.

### (ii) Secondary metabolism (5 genes).

Disruption of genes involved in chorismate synthesis, including *aroB*, *-C*, *-D*, *-E*, and *-K*, turned out to be deleterious during colonization of epithelial and endothelial cell monolayers. Chorismate is the common branch point for the production of a wide array of metabolites, such as aromatic amino acids (phenylalanine, tryptophan, and tyrosine), vitamin K, vitamin E, coenzyme Q, folate, enterobactin, plastoquinones, and phenoxazinones.

### (iii) Transporter proteins (16 genes).

Our screen also identified genes implied to belong in the phosphoenolpyruvate:carbohydrate phosphotransferase system (PTS), a transport system for sugars and sugar derivatives. In particular, transposon-induced disruption of *pts I*, which encodes the phosphotransferase enzyme I, and *ptsH*, known to encode the phosphocarrier protein HPr, had detrimental effects on colonization of human cells (31). Other transporters were also found to be important for colonization of both epithelial and endothelial cells (see Table S4 in the supplemental material), such as 3 secondary transporters of the ABC family (*ftsX*, NMA1811, and NMA0414).

### (iv) Cell motility (12 genes).

It was not unexpected that genes of the type IV pilus machinery were found to be necessary for colonization of both cell types (*pglD* and *pilC2*, *-D*, *-E*, *-M*, *-N*, *-P*, and *-Q*). In addition, three pseudopilin genes, namely, *pilS2*, *-S4*, and *-S5*, which are involved in colonization, were selected negatively in both *in vitro* models. On the other hand, disruption of *pilT*, which abolishes pilus retraction, was selected positively in both cell types.

### Analysis of genes important for sole colonization of epithelial cells.

Among the 288 genes important for host cell colonization, 151 were uniquely selected during colonization of epithelial cells (see Fig. S2A in the supplemental material). The vast majority were related to metabolism. Of note, the *glk* gene is responsible for transformation of d-glucose into d-glucose-6P and allows the start of glycolysis or its alternative, the KDPG pathway. *N. meningitidis* has all the genes coding for the TCA cycle except for the malate dehydrogenase gene (32), but it has an alternative subpathway where the enzyme malate:quinone oxidoreductase (Mqo) synthesizes oxaloacetate from (*S*)-malate (quinone route), like *Helicobacter pylori* (33). Although this enzyme is not essential for *N. meningitidis* to grow, it is important for colonization of epithelial cells (see Table S3 and Fig. S2A in the supplemental material).

Our screen identified two enzymes involved in galactose metabolism, *galE* and *galE*′. In particular, the *galE* gene product plays an essential role in the incorporation of galactose into meningococcal lipooligosacharide surface molecules, which are important for pathogenesis (34).

Other enzymes involved in biosynthesis of amino acids, such as *argA*, *-G*, and *-J* and *ilvC*, *-D*, *-E*, and -*I*, and *lysA* were also selected as necessary for colonization of epithelial cell monolayers, together with enzymes involved in DNA mismatch and repair (*XSeb*, *mutS*) and in DNA replication (*rnhB*). The 50S ribosomal protein L7/L12 encoded by *rplL* forms part of the ribosomal stalk, which favors the interaction of the ribosome with GTP-bound translation factors. Although it should be essential, like the rest of 50S ribosomal proteins, for accurate translation we found that it was only necessary for colonization of the epithelial layer.

Interestingly, all the genes of the operon *mtrCDE* were negatively selected on epithelial cells, whereas the transcriptional negative regulator *mtrR* was positively selected (see Table S3 in the supplemental material). This operon encodes the Mtr efflux pump responsible for *Neisseriaceae* resistance to penicillin and antimicrobial peptides (35). Moreover, it has been hypothesized that such efflux systems may enable mucosal pathogens like gonococci to resist endogenous antimicrobial peptides that are thought to act during infection, similar to LL-37, which is produced by epithelial cells (36).

### Analysis of genes important for sole colonization on endothelial cells.

Twenty-nine transposon-disrupted genes were identified to be specific for adhesion to endothelial cells (see Fig. S2B and Table S3 in the supplemental material). Four of them were involved in the respiratory electron chain: *nuoB*, *-E*, and *-I* from the oxidative phosphorylation complex I, and *ppk*, which catalyzes the reversible transfer of the terminal phosphate of ATP to form a long-chain polyphosphate (polyP). Genes involved in amino acid metabolism were also important, including *aroA* and *-G* and *trpG*, which are involved in phenylalanine, tyrosine, and tryptophan biosynthesis, and *purA* and *-F* and NMA1684, which are important for alanine, aspartate, glutamate, and purine metabolism. Interestingly, only three type IV pilus genes, *pilJ*, *-K*, and *-O*, were significantly important for colonization of endothelial cells, as well as genes for two sodium transporters, NMA1901 and NMA2083, which are a putative amino acid symporter and a sodium/proline symporter (proline permease), respectively.

### Identification of intergenic regions containing sRNA important for colonization of human cells.

IRs containing sRNA candidates were also checked by using Tn-seq for conditional essentiality for colonization of epithelial and endothelial cells. As reported previously, we arbitrarily considered important for cell colonization the sRNA-associated IRs with a log_2_ FC less than −1.4 and an adjusted *P* value of <0.05, while those not required for cell colonization were sRNA-associated IRs with a log_2_ FC greater than 1.4 with an adjusted *P* value of <0.05. Thus, a total of 33 IRs containing sRNAs were necessary, from which 5 were common to both human cell types, and 18 and 5 genes were specifically selected during colonization of epithelial and endothelial cells, respectively (see Table S5 in the supplemental material). On the other hand, a total of 33 IRs containing sRNAs were found to be beneficial, from which 27 and 6 were only selected after passage on epithelial and endothelial cells, respectively.

## DISCUSSION

Here, we have taken advantage of a high-throughput whole-genome screen to determine meningococcal genes involved in human cell colonization. Our exhaustive genetic screen of the *N. meningitidis* Z5463 genome has first allowed the identification of 383 genes essential for growth, representing 19% of the bacterial genome, and 329 genes having a growth defect, representing 16% of the genome. The nonessential genes were then studied for their role during cell colonization. Among these genes, 288 have been found to be necessary for colonization of human epithelial and/or endothelial cells, suggesting that *N. meningitidis* has developed dedicated tools to efficiently colonize human cells.

Transposon insertions within an operon may exert a polar effect on downstream genes, resulting in decreased expression of a downstream gene(s). However, the transposon insertion itself does not disrupt transcription of an upstream promoter. Indeed, we observed 84 such genes in operons without a noticeable effect on gene essentiality, thus confirming that a transposon insertion does not induce a polar effect.

Recent studies on sRNAs have demonstrated that they are key elements of posttranscriptional gene regulation in bacteria (19, 37). Although *N. meningitidis* is able to adapt to different host niches during human infection, only a few sRNAs have been fully described to date. Recently, transcriptional expression profiling of *N. meningitidis* strain MC58 in human blood *ex vivo* revealed 91 differentially expressed putative sRNAs (38), and this list was enlarged to up to 98 sRNAs *in vitro* by Fagnocchi and coworkers (19). Among these 98 sRNAs, 68 were located in IRs between two annotated ORFs. Here, we combined our data from Tn-seq with RNA sequencing to confirm the expression of sRNAs in IRs having a regulatory function over gene expression. A total of 390 sRNAs located in IRs were identified by RNA sequencing, of which 30 were located in IRs already described by Fagnocchi and coworkers to contain sRNAs. We did not find all the sRNAs characterized earlier by Fagnocchi et al. Two reasons can explain this discrepancy: (i) we used a different meningococcal strain, and (ii) our experimental conditions were different. While we only verified gene under mid-log-phase growth conditions, the other authors performed transcriptional expression profiling of *N. meningitidis* after exposure of the bacterium to stress signals (e.g., heat shock, oxidative stress, iron and carbon source limitation), thus identifying putative sRNAs differentially expressed *in vitro*.

### Analysis of essential genes.

The essential metabolic routes are the pentose phosphate and the KDPG pathways, which are responsible for glucose catabolism and oxidative phosphorylation, together with pathways involved in the synthesis of nucleotides, amino acids, vitamins, lipids, lipooligosaccharide, and peptidoglycan.

Iron uptake systems are major players for *N. meningitidis* pathogenesis (39, 40). Meningococci have developed 3 mechanisms to extract iron from its human host: (i) the transferrin and lactoferrin receptors, (ii) the hemoglobin receptor (*hmbR* or *hpuB2*), and (iii) the haptoglobin-hemoglobin receptor, also called the heterodimeric HpuAB complex. In our study, where iron was present as ferric nitrate, the ferric uptake transcriptional regulator *fur* was found to be essential and the lactoferrin-binding protein encoded by *lbpB* was found to be growth defective, together with *hpuB* and *fetA*, a TonB-dependent enterobactin receptor. In addition, two ABC transporters, namely, NMA0451 and NMA0577, which are involved in the putative ferric enterobactin uptake system, were found to be essential, whereas two other putative ferric enterobactin proteins turned out to results in a growth defect, namely, NMA0448 and NMA0450. Although it has been shown that *N. meningitidis* requires a *ton* system for utilization of transferrin, lactoferrin, hemoglobin, and haptoglobin-hemoglobin (41, 42), the proteins TonB, ExbB, and ExbD that form the TonB complex were not found to be essential in our screen.

Our results are consistent with the previous system-wide approach carried out by Mendum et al. (3) (see Table S6 in the supplemental material). Interestingly, despite differences observed for single gene requirements between both studies, essential metabolism pathways are very similar regardless of the growth medium (see Fig. S1 in the supplemental material). The main differences observed concern the need for synthesis of cofactors and vitamins on GC broth (GCB) agar compared to requirements for growth on other media. These discrepancies are likely due to differences in metabolite profiles between each medium.

The list of *N. meningitidis* essential genes was further compared to essential genes of other organisms listed in the Database of Essential Genes (DEG) (43), which lists bacterial genes essential for viability in different species. From the 383 essential genes determined in our study, 29% had essential orthologs with *Escherichia coli* (43, 44), 26% with *Haemophilus influenzae* (45), 48% with *Pseudomonas aeruginosa* (46), and 55% with *Salmonella enterica* serovar Typhi (47) (see Table S6 in the supplemental material). All these Gram-negative bacteria shared 77 core essential genes that were involved in information storage and processing, as well as cellular processes, metabolism, and lipooligosaccharide synthesis (see Table S7 in the supplemental material), providing a core essential genome for Gram-negative pathogens.

We compared our list of genes that caused defects in growth and of essential genes with that from the library of Rusniok and coworkers, which contained 947 mutated genes of *N. meningitidis* M8013 (serogroup C) (48). The 824 genes that had a correspondence with our strain Z5463 are listed in Table S7 of the supplemental material. We found that 11% of those genes encoded proteins that caused growth defects in our analysis, and 7% were essential. This confirms that the large majority of our core essential genes are indeed essential for *N. meningitidis* survival. Moreover, our genetic screen is the result of a competition between different mutants harboring a transposon insertion for a single gene within a mixed population, thus explaining some possible discrepancies between the two studies. For example, the *fur* gene was classified by us as essential, whereas a deletion mutant has been proven to be viable despite having an important growth defect (49).

We further compared our list of genes that caused growth defects and of the essential genes to the minimal gene set created by Gil and coworkers (50), which can be found in the publicly accessible thematic database NeisseriaScope within MicroScope (51). The minimal gene set within NeisseriaScope includes well-conserved housekeeping genes for basic metabolism and macromolecular synthesis, many of which are essential. As expected, our data indicated that among the minimal gene set for *N. meningitidis*, 50% corresponds to essential genes and 20% to genes associated with growth defects (see Table S7).

### Analysis of genes involved in cell colonization.

In recent years, the concept of nutritional virulence has shown increasing significance for explaining various metabolic adaptations that successfully exploit available host nutrients for pathogen proliferation. For instance, Schoen et al. recently compiled a list of “omics” approaches for metabolic adaptation of meningococci upon adhesion to human cells and for growth in human blood (52), and thus they illustrated how the metabolism of lactate, the oxidative stress response, glutathione metabolism, and the denitrification pathway are linked to meningococcal pathogenesis. Among the 288 genes identified to be important for colonization of human epithelial or endothelial cells, 108 genes were necessary for colonization of both human cell types, whereas 151 and 29 were only selected in the epithelial or endothelial cell model, respectively. The majority of these genes were involved in metabolic pathways.

**FIG 5:**
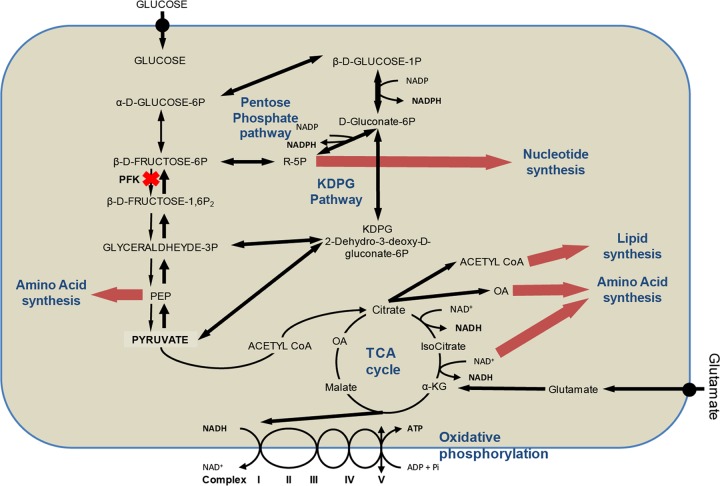
Schematic representation of the main metabolic pathways of conditional essential genes in *N. meningitidis* necessary for colonization of both epithelial and endothelial cells. The illustration shows the main metabolism pathways in proliferating cells, including glycolysis, TCA cycle, PP, KDPG pathways and the synthesis of nucleotides, lipids, and amino acids. Black bold arrows indicate the metabolism reorientation, whereas red bold arrows indicate the main biosynthesis pathways important during colonization.

For both cell types, a metabolic reorientation toward the production of β-d-fructose-6P and PEP was suggested by our Tn-seq analysis. Indeed, several genes involved in the gluconeogenic route, such as *fba* and *fbp*, which lead to the production of β-d-fructose-6P, were identified, as well as two genes that lead to the production of PEP (*pykA* and *ppc*). β-d-Fructose-6P and PEP are important branch points that connect to the PP, KDPG, and nucleotides/amino acid synthesis pathways. One gene involved in the metabolism of galactose (*galE*) and one gene involved in d-glucose metabolism (*glk*) are also important for cell colonization, confirming that glucose consumption is critical for growth on cells. Furthermore, *N. meningitidis* switches its metabolism toward biosynthesis of cellular components. This recapitulates what has been described in proliferative cancer cells, where the high abundance of glucose is metabolized through the PP pathway to produce nucleosides and NADPH, which is essential for fatty acid synthesis (Fig. 5). NADPH also contributes to better redox control by reducing the amount of reactive oxygen species via glutathione metabolism (53). Interestingly, we found a gene, NMA0486, that is important for l-glutamate uptake from the environment. This gene was negatively selected for colonization of epithelial cells. It has been shown that l-glutamate uptake by this transporter enhances the production of glutathione, resulting in increased meningococcal survival (54) and thus confirming the important contribution of the antioxidant effect of glutathione.

Concerning cell adhesion genes, almost all type IV pilus genes were identified to be important except for *pilT*, which was not necessary. The *pilC* gene, known to play a key role in type IV pilus biogenenesis and cell adhesion (55, 56), was also found to be important for colonization of both human cell types, although it did not reach our threshold of a log_2_ FC less than −1.4 in endothelial cells. Interestingly, only *pilJ*, *-K*, and *-O* were important for adherence to endothelial cells, and three pseudopilin genes, namely, *pilS2*, *-S4*, and *-S5*, were negatively selected. Furthermore, Deghmane et al. showed that *crgA* (NMA0601)-encoded transcriptional factor, which was found in our study to be important for colonization of epithelial cells, is involved in cell colonization through the modulation of PilE and capsule expression (57). In addition, one of the four capsule biosynthesis genes, *sacC*, was found to be important for the colonization of both human cell types. The fact that none of the other genes involved in capsule biosynthesis was found to be important for colonization suggests that *sacC* may have an additional cellular function.

Of the five mutants identified by Jamet et al. in a genome-wide screen (13), we confirmed the importance of *narP* (NMA1419) in human epithelial cell colonization. NarP is a transcriptional regulator of the two-component system NarP/NarQ, which regulates the availability of nitrite in the cell (13, 23). Here, two genes involved in the denitrification pathway, *aniA* and *norB*, were also found to be essential for growth. This pathway, described by Rock et al., allows meningococcal respiration when oxygen is restricted (23), thus suggesting that oxygen was limited under our culture conditions. This pathway first allows nitrite (NO^2−^) to be reduced to nitric oxide (NO) by the copper nitrite reductase AniA. Then, NO is further reduced to nitrous oxide (N_2_O) by the quinoloxidizing nitric oxide reductase NorB. Our data confirm the role of the nitrite reduction pathway in bacterial growth.

In summary, we have provided here a comprehensive analysis of the genes required for *N. meningitidis* growth and colonization of human endothelial and epithelial cells *in vitro*. Moreover, the transposon libraries constructed in this work represent a relevant tool that may serve to further investigate meningococcal pathogenesis in different environments Deciphering new insights into the metabolic adaptations of *N. meningitidis* during pathogenesis will help efforts to fight this human pathogen more efficiently.

## MATERIALS AND METHODS

### Bacterial strains and growth conditions.

*N. meningitidis* Z5463, a naturally transformable capsulated serogroup A strain, was used to generate saturating Tn insertion mutant libraries. Z5463 belongs to the same sequence type (ST) as strain Z2491, i.e., ST-4 (58), subgroup IV-1, expressing OpaA and OpaC and in the same clonal group as Z2491, thus allowing the use of the genomic sequence of the latter strain. Because *N. meningitidis* Z2491, whose genome has been completed by the Sanger Center (20), is not transformable, we routinely work with strain Z5463 (59). A comparison between both strains has been made in the public databases for molecular typing and microbial genome diversity (PubMLST), and it has been found that both strains have 168 single nucleotide polymorphism (SNP) differences, and only two genes are present in one strain and not in the other: loci NEIS2357 and NEIS2494. The variability in the 168 loci found in both strains is not an issue, since a 10% mismatch is allowed for the Tn-seq read mapping. Z5463 was stored frozen at −80°C and was routinely grown at 37°C in 5% CO_2_ in GC liquid medium under shaking conditions or on GC medium base (Difco) containing Kellogg’s supplements (60). Kanamycin was used at a concentration of 200 µg/ml.

### Endothelial and epithelial cell culture conditions.

The immortalized human brain endothelial cell line (hCMEC/D3) (61), which retains the main characteristics of primary brain endothelial cells, was grown and infected as described by Coureuil et al. (62). The pharynx carcinoma-derived FaDu epithelial cells were grown and infected as described by Jamet et al. (13). Laminar flow chamber experiments were performed as described by Jamet et al. (13). Disposable flow chambers composed of six independent flow channels (μ-Slide VI 0.4; ibidi) were used. hCMEC/D3 or FaDu cells were seeded at a density of 0.3 × 10^5^/cm^2^ and incubated for 7 days at 37°C in 5% CO_2_ until confluent. Examination of the cell layers was performed before each flow assay, and only channels with a uniformly confluent layer were used.

### Transposon mutant library construction.

Construction of mutant libraries was performed using the Template Generation System II kit (Thermo Scientific), which includes Entranceposon KanR-3, a derivative of the bacteriophage mu containing a kanamycin resistance marker. Briefly, genomic DNA from *N. meningitidis* Z5463 was extracted by using chloroform and then ethanol precipitated. *In vitro* transposition reactions were carried out with 0.88 µg of MuA transposase, 80 ng of Entranceposon KanR-3, and 5 µg of *N. meningitidis* Z5463 genomic DNA. Reaction mixtures were incubated for 3 h at 30°C following the manufacturer’s instructions and ethanol precipitated. Purified transposition reaction products were transformed into naturally competent *N. meningitidis* Z5463 as described previously (63, 64) and selected on GCB agar plates containing 200 µg of kanamycin per ml. On the following day, transformants were counted with an eCount colony counter (Heathrow Scientific), scraped off of agar plates, and stored at −80°C in GCB supplemented with 20% glycerol. When a large amount of transformants was reached, they were pooled into libraries of approximately 70,000 CFU per library. To separate transformants from any residual agar, cells were grown in GCB for a maximum of 2 h, after which libraries were collected and stored at −80°C in GCB supplemented with 20% glycerol (Fig. 1A). We obtained three independent libraries of approximately 70,000 transformants each.

### *In vitro* screening of Tn libraries for colonization.

The HITS technology was used to profile the relative abundance of each mutant in all libraries after selection in the *in vitro* laminar flow chamber experimental model, as described previously (11, 13). When Tn mutant libraries were assayed, an aliquot of the library was thawed and grown in hCMEC/D3 or FaDu CCM until mid-log phase (exponential phase of growth). The cultured Tn libraries were adjusted to an optical density at 600 nm of 0.5 in the same culture medium, and 60-µl aliquots (corresponding to approximately 3 × 10^7^ bacteria) was used to inoculate 6 channels of a flow chamber containing an endothelial or epithelial cell monolayer. The remaining culture of the Tn library is referred to as the input pool. Bacteria were allowed to adhere to endothelial or epithelial cells for 1.5 h without flow. At 1.5 h postinfection, a continuous flow of CCM containing 3 µg of vancomycin/ml was applied for 18 h at a constant flow rate of 0.04 ml/min by using a syringe pump (Harvard Apparatus). The flow chamber was placed in an incubator at 37°C with 5% CO_2_ throughout the experiment. After 18 h, the recovered bacteria, (i.e., the bacteria obtained from aspiration of the 6 channels and constituting the output pool) were harvested in a microcentrifuge tube by centrifugation. Bacterial pellets were resuspended in a lysis solution (40 mM Tris-acetate [pH 7.8], 20 mM sodium acetate, 1 mM EDTA, 1% SDS). Chromosomal DNA extraction was performed using chloroform followed by ethanol precipitation for both the input and output mutant pools (Fig. 1B).

### Identification of transposon insertion sites.

A strategy of capture by hybridization combined with next-generation sequencing (Illumina technology) was used to identify transposon insertion sites. The procedure is similar to that described by Depledge et al. (65) to identify virus insertion positions in the human genome, except that a unique biotinylated oligonucleotide specific to the transposon extremities was used as bait to capture transposon-containing bacterial fragments (see Text S1 in the supplemental material for further details). DNAs from input and output pools of epithelial library 1 (InEpi.1A and OutEpi.1A) were first sequenced in a single flow cell lane and yielded ~18 and ~20 million raw reads, respectively (Table 1). Then, input and output pools of epithelial libraries 1 to 3 were sequenced together by using a single flow cell lane and yielded between ~6 and ~10 million raw reads per sample (Table 1). Likewise, the 6 endothelial libraries (InEndo.1 to -3 and OutEndo.1 to -3) were sequenced together in a single flow cell lane and yielded between ~7 and ~10 million raw reads per sample (Table 1).

Libraries enriched in transposon-containing bacterial fragments were sequenced on an Illumina MiSeq (paired-end sequencing of 300 plus 300 bases, from 2 to 6 samples per run).

### Bioinformatic analysis of Tn libraries.

HITS data analysis was performed as described previously (14), with minor modifications. The detailed procedure is described in Text S1 in the supplemental material. Gene essentiality was determined based on the log_2_ of the measured number of transposon-containing reads per gene divided by the expected number of transposon-containing reads per gene (based on the number of possible transposon insertion sites per gene, the mutant library size, and the sequencing depth) as determined via TMM normalization (see Table S1A in the supplemental material). Determination of the log_2_ FC for identification of conditional essential genes was performed with the binary logarithm of the number of reads of the target sample (output libraries harvested after selection onto epithelial or endothelial cells, respectively) divided by the number of reads of the gene within the control sample (input libraries grown before selection in epithelial or endothelial cells, respectively) (see Table S1D). 

### Web tools used for analysis.

Putative orthologs of *N. meningitidis* genes were identified by using the DEG database (http://tubic.tju.edu.cn/deg/) (43). The protein families in DEG corresponded to homologous ORFs with identical assigned functions.

Metabolic pathways and subsystems for *N. meningitidis* strain Z2491 were obtained based on Kyoto Encyclopedia of Genes and Genomes orthology (66). The list of transporter proteins was obtained from data available from the Transporter Protein Analysis Database (TransportDB) at http://www.membranetransport.org.

### RNA sequencing. (i) Isolation of bacterial RNA.

Bacteria grown in Ham F-12 medium (PAA Laboratories) supplemented with 10% fetal calf serum (FCS; PAA Laboratories), 20 mM HEPES (PAA Laboratories) at 37°C in a humidified incubator under 5% CO_2_ and under shaking conditions to the mid-logarithmic phase were harvested by centrifugation. Bacterial pellets were resuspended in 1 ml of TRIzol reagent (Life Technologies) and frozen at − 80°C. RNA isolation was performed according to TRIzol RNA isolation procedure. Quality of the bacterial RNA was measured using a Bioanalyzer 2100 (Agilent). To remove contaminating genomic DNA, samples were treated with 0.25 U of DNase I (Fermentas) per µg of RNA for 45 min at 37°C.

Preparation of 3 whole transcriptome libraries and 3 sRNA enriched libraries as well as the RNA-Seq experiments are detailed in supplemental Text S1 in the supplemental material*.*

### (ii) Bioinformatic analysis of expression data.

Totals of 75.2 million reads and 2.1 million reads were obtained from the whole-transcriptome libraries and the sRNA-enriched libraries, respectively. Reads matching ribosomal genes based on SortMeRNA (67) and low-quality reads were removed using Trimmomatic (parameters: leading, 8; trailing, 10; sliding window, 4:5) (68). The remaining sequences were independently mapped with Bowtie version 0.7.12 (69) to the reference sequence of *Neisseria meningitidis* Z2491 (accession number AL157959.1). These data are presented in Table S2 in the supplemental material.

The expression of mRNA was measured by extracting the read counts with HTSeq (70) and transformed into RPKM values for each replicate. We arbitrarily defined the cutoff for classification of a genomic region of interest as the median of RPKM values of intergenic regions (see Table S2D to F in the supplemental material). Thus, a genomic region was considered transcribed if it had an RPKM value greater than 2.3. Out of a total of 1,994 CDS (not including rRNAs or tRNAs), 1,831 were above this transcriptional threshold (92%), whereas 163 CDS were below (8%) and thus not expressed under these experimental conditions. Alignment files were then used to detect putative sRNAs with the help of sRNA-Detect (71). That tool allowed us to detect 6,088 putative sRNAs in the whole-transcriptome data set and 4,530 putative sRNAs in the sRNA enriched data set. The set of sRNA candidates provided by sRNADetect from the two data sets were combined. Next, genes flanking each sRNA candidate were extracted from the annotation for *N. meningitidis*, and only sRNA candidates with a clear assignment to a particular strand, requiring that at least 99% of the reads originated from the plus or minus strand, were kept. When an intergenic region contained more than one sRNA, the candidate with the highest coverage was selected. The average coverage of the sRNA and the flanking genes was next calculated by using bedtools (72) for each strand and by using customized Python scripts. In order to analyze whether the sRNAs overlapped a promoter region, the tool PromBase (73) was used, and putative promoter regions were annotated in the genome of *N. meningitidis* (see Table S2B). 

### Nucleotide sequence accession numbers.

RNA-seq data are available in the ArrayExpress database under accession number E-MTAB-4768. The transposon sequence reads we obtained have been submitted to the ENA database under accession number PRJEB11986.

## SUPPLEMENTAL MATERIAL

Text S1  Supplemental methods and references. Download Text S1, DOCX file, 0.04 MB

Figure S1  Metabolic overview of genes that contribute to *N. meningitidis* growth in GCB agar and CCM. Download Figure S1, TIF file, 1.3 MB

Figure S2  Schematic representation of the main metabolic pathways of conditional essential genes necessary for colonization of epithelial and endothelial cells. Download Figure S2, TIF file, 0.2 MB

Table S1  Tn sequencing raw analysis output obtained from ESSENTIALS tool kit. Table S1, XLSX file, 1.6 MB

Table S2  RNA-seq data. Table S2, XLSX file, 0.7 MB

Table S3  Core essential genes and genes important for colonization of epithelial and endothelial cells. Table S3, XLSX file, 0.2 MB

Table S4  Transporters and relevant pathways. Table S4, XLSX file, 0.1 MB

Table S5  Tn sequencing raw analysis output obtained from ESSENTIALS tool kit for sRNA-associated intergenic regions (IR) conditional essentiality. Table S5, XLSX file, 0.2 MB

Table S6  Essentiality predictions for *N. meningitidis* and other Gram-negative bacteria. Table S6, XLSX file, 0.01 MB

Table S7  Gene essentiality comparison. Table S7, XLSX file, 0.2 MB
