# Supplementary material for: Comprehensive Identification of Meningococcal Genes and Small Noncoding RNAs Required for Host Cell Colonization
Source: mBio. 2016 Aug 2;7(4):e01173-16. doi: 10.1128/mBio.01173-16 (PMC4981724; doi:10.1128/mBio.01173-16)
Supplement: Text S1 — Supplemental methods and references. Download [file mbo004162920s1.docx]

**SUPPLEMENTAL MATERIAL**

**SUPPLEMENTAL MATERIAL AND METHODS**

**Identification of transposon insertion sites**

A strategy of capture by hybridization combined to next generation sequencing (Illumina technology) was used to identify transposon insertion sites in bacterial DNA. The procedure is similar to the one used by Depledge *et al* (1) to identify virus insertion position in the human genome, except that a unique biotinylated oligonucleotide specific to the transposon extremities is used as a bait to capture transposon containing bacterial fragments. First, Illumina compatible precapture barcoded DNA libraries were constructed using the Ovation Ultralow kit (Nugen Technologies) as recommended by the manufacturer. One to 3 µg of each DNA was mechanically fragmented to a median size of 200 bp using a Covaris (model E220). One hundred ng of double strand fragmented DNA was end-repaired and adaptors containing a specific 8 bases barcode were ligated to the repaired ends (one specific barcode per DNA sample). After a purification step, the ligated DNA fragments were PCR amplified (7 cycles) to get the final precapture barcoded libraries. A multiplex capture was performed on an equimolar pool of these barcoded libraries. Briefly, 500 ng of this heat denatured pool of libraries and 2 µl of 1.5µM biotinylated oligonucleotides specific to the transposon (5’–Biotin/ACA ACG TGG CTT ACT AGG ATC CGT TTA AAC GGT GGC TGG AGT TAG ACA TCG TTT TCG CAT TTA TCG TGA AAC GCT TTC GCG TTT TTC GTG CGC CGC TTC A -Phosphate/-3’, Integrated DNA Technologies) were hybridized for 16 to 24 hrs using SureSelect hybridization reagents and protocol (Agilent Technologies). The hybrids formed between the barcoded libraries molecules complementary to the biotinylated beads were captured according to the Agilent SureSelect protocol using streptavidine coated magnetic beads and a magnet. The captured libraries molecules were PCR amplified (15 to 16 cycles) to generate a final pool of postcapture libraries. Postcapture libraries, enriched in transposon containing bacterial fragments, were sequenced on an Illumina MiSeq (Paired-End sequencing 300+300 bases, from 2 to 6 samples per run).

**Bioinformatic analysis of Tn libraries**

HITS data analysis was performed as described previously, with minor modifications. Briefly, the Illumina sequencing reads were filtered on phred30 quality and 150 bp length using the fastx toolkit (2). Reads that contained the Entranceposon KanR3 inverted terminal repeat (ITR) sequence were identified in the raw fasta files of the HITS results and trimmed of the Entranceposon sequence via the cutadapt tool (3). Reads shorter than 15 bp were removed. Transposon containing reads were processed using the ESSENTIALS software with default settings. Processed sequence reads of HITS libraries, typically 50 bp in length, were mapped on the genome of *N. meningitidis* Z2491 (4). Count data (readcounts) were generated per unique sequence read or per gene and corrected by locally weighted scatterplot smoothing (LOESS) for the bias in HITS data caused by the increase in available DNA close to the origin of replication (ORI). Normalization factors were calculated using trimmed mean of M values (TMM). Transposon containing reads in the control and target samples were tested for significant differences (adjusted p-value < 0.05) by the Cox-Reid paired test implemented in EdgeR (5) assuming moderated tagwise dispersion of replicates. The prior n value to determine the amount of smoothing of tagwise dispersions was set at 5. *P* value adjustment (adjusted p-value < 0.05) was based on the Benjamini-Hochberg procedure (5).

Preparation of cDNA libraries and RNA-Seq

RNA-Seq analyses were performed in *N. meningitidis* strain Z5463 by deep sequencing using Ion Torrent technology (Thermo Fischer Scientific). For whole transcriptome libraries (mRNA+sRNA), cDNA libraries were prepared using the Ion Total RNA-Seq Kit v2 (Life Technologies) including a prior step of ribodepletion using specific probes adapted to *N. meningitidis* (Low Input Ribo Minus Eukaryote System v2) and size fractionation of RNA prior to cDNA synthesis with RNase III was performed. Briefly, 500 ng of total RNA were ribodepleted and then fragmented. After fragmentation, mean size of fragment was around 120 nt. For sRNA libraries (sRNA), three cDNA libraries were prepared using the Ion Total RNA-Seq Kit v2 for Small RNA Libraries (Life Technologies) including a prior step of enrichment in small RNA fraction. Equal amounts of total RNA were used for the generation of all cDNA libraries. The three whole transcriptome libraries (mRNA+sRNA) were then sequenced on an Ion Proton^TM^System (Single-End sequencing) using the Ion PI Template and Sequencing OT 200 kit v3 and the 3 sRNA libraries were sequenced on an Ion PGM (Single-End sequencing) with the Hi-Q Template and Sequencing Kit using a 316 v2 array.

**SUPPLEMENTAL FIGURES**

**Figure S1: Metabolic overview of genes contributing to *N. meningitidis* growth in GCB agar and CCM.** (A) Black bars indicate the essential genes and grey bars indicate the growth defective genes on GCB agar and CCM found in our study. Dots indicate metabolic products. All maps were obtained from the Kyoto Encyclopedia of Genes and Genomes orthology (Kanehisa *et al.* 2012). (B) Comparison of the essential genes for growth in minimal medium from our study and Mendum’s study. Red bars depict essential genes obtained by Mendum *et al* 2011 grown on minimal medium. (C) Comparison of the essential genes for growth in serum from our study and Mendum’s study. Red bars depict essential genes obtained by Mendum *et al* 2011 grown on serum. (D) Comparison of the essential genes for growth in CAB medium (rich blood-based medium) from our study and Mendum’s study. Red bars depict essential genes obtained by Mendum *et al* 2011 grown on CAB medium.

**Figure S2: Schematic representation of the main metabolic pathways of conditional essential genes necessary for colonization of epithelial and endothelial cells.** (A) The genes necessary for epithelial cells colonization (log_2_ FC <-1.4; adj. P value<0.05) are indicated in red whereas the beneficial ones (log_2_ FC >1.4; adj. P value<0.05) are indicated in green. *1: *ilvC/D/E/I*; *2: *argA/G/J* *; *3: sucC/D; *4: nuoM; *5: sdhB/D; *6: atpC*. (B) The genes necessary for endothelial cells colonization (log_2_ FC <-1.4; adj. P value<0.05) are indicated in red whereas the beneficial ones (log_2_ FC >1.4; adj. P value<0.05) are indicated in green. *1: *aroA/G and trpG*; *2: ilvA*; *3: ilvE* and *leuA; *4:* NMA1684*; *5: nuoB/E/I*.

**SUPPLEMENTAL TABLES**

**Table S1: Table with Tn-sequencing raw analysis output obtained from Essentials toolkit**

This table contains information regarding the output files from the Essentials toolkit. For the different genomic features, it gives information about the location and function of CDS and the number of unique transposon insertion site flanking sequences.

(A). Gene essentiality output file. Column B contains the locus tags, columns marked with .x contain the data before between-sample normalization. The final .x column contains the number of unique insertion site flanking sequences. Column marked with .y contain data normalized (pseudocounts). The final .y column contains the expected number of reads. The next four columns contain data generated by the statistical test. logConc: The signal calculated as the log fraction of the number of reads of a gene divided by the total number of reads, logFC: log_2_ of the number of reads of the target sample divided by the number of reads of the control sample. P-value: probability of obtaining a test statistic as the one that was actually observed. Adj. P val: P value adjusted for multiple testing. The final seven columns contain information about the location, size and annotation of the gene. Genes marked with NA have less than 2 reads per sample and less than 2 insertion sites assigned to them because the gene has no unique insertion sites or because the gene is very small. These genes are excluded from the analysis. The last column contains the average mRNA expression in RPKM from replicates 1 to 3. Genes colored in grey are below the expression threshold of 2.3, thus not being expressed. (B) Rarefaction analysis of separate Tnseq libraries and all libraries combined. Insertion sites were randomly selected with increments of 50. Genes that had a transposon insertion were counted. The number sites sampled was plotted against the number of genes hit. Although complete saturation has not been reached, increasing the size or number of the mutant libraries would only result in a very minor increase in genes hit by the transposon. (C) Poisson’s derivation calculation. (D) Conditional gene essentiality output. Column B contains the locus tags, columns marked with .x contain the data before between-sample normalization. Columns marked with .y contain data normalized (pseudocounts). The next eight columns contain information about the location, size and annotation of the gene. The final fourteen columns contain data generated by the statistical test. logConc: The signal calculated as the log fraction of the number of reads of a gene divided by the total number of reads, log_2_FC: log_2_ of the number of reads of the target sample divided by the number of reads of the control sample. P-value: probability of obtaining a test statistic as the one that was actually observed. Adj. P val: P value adjusted for multiple testing. Genes marked with NA have less than 10 reads per sample assigned to them either because they are essential, because they are not hit in the mutant library, or because the gene has no unique insertion sites. (E) sRNA associated intergenic regions essentiality output file. This table has been built like tab A but in this case column B contains the name of the intergenic regions containing an sRNA candidate (sRNAaIR). (F) Poisson’s derivation for the sRNA associated intergenic regions.

**Table S2: RNAseq data**

This table contains the results concerning the RNA sequencing. (A) Read overview. (B) Coverage of sRNA. (C) Summary of sRNA associated intergenic regions (sRNAaIR). (D) mRNA expression. (E) Intergenic regions expression. (F) Summary of mRNA expression. (G) Comparative analysis sRNAs with Fagnocchi *et al* study (6).

**Table S3: Core essential genes and genes important for colonization of epithelial and endothelial cells**

The following table contains information related to the significant log_2_FC values with an adjusted p-value <0.05 for the essential and growth defective genes, as well as for the genes important for colonization of epithelial and endothelial cells. Each tab contains positive or negative values either for both selection models, *in vitro* selection in epithelial (EPI) or endothelial (ENDO) cells, or for common (COMMON) genes selected equally in both systems.

**Table S4: Transporters and relevant pathways**

This table contains information regarding transporter proteins and relevant pathways either essential or growth defective, as well as important for colonization of epithelial and endothelial cells.

**Table S5: Table with Tn-sequencing raw analysis output obtained from Essentials toolkit for sRNA associated IR conditional essentiality**

This table contains information regarding the output files from the Essentials toolkit for the sRNA associated IR conditional essentiality for colonization of the both human cell types. Column A contains the name of the intergenic regions containing an sRNA candidate (sRNAaIR). The next columns contain data generated by the statistical test. logConc: The signal calculated as the log fraction of the number of reads of a gene divided by the total number of reads, log_2_FC: log_2_ of the number of reads of the target sample divided by the number of reads of the control sample. P-value: probability of obtaining a test statistic as the one that was actually observed. Adj. P val: P value adjusted for multiple testing.

**Table S6: Essentiality predictions for *N. meningitidi*s and other Gram-negative bacteria.**

This table displays the number of essential genes identified in our study and other studies with Gram-negative bacteria.

**Table S7: Gene essentiality comparison**

This table contains different comparison analysis between the essential genes identified in our study and other studies. (A) Tables A1 to A4 display the core essential genes common to 5 Gram-negative bacteria: *N. meningitidis* Z2491, *E. coli*, *H. influenzae*, *P. Aaeruginosa* PAO1, *S. enterica* serovar typhi. (B) Tables B1 to B4 display the data from the Rusniok *et al* study (7) compared to ours. (C) This table displays the minimal gene set of *N. meningitidis* compared to the minimal gene set taken from Gil and co-workers (8).

**SUPPLEMENTARY MATERIAL REFERENCES**

1. **Depledge DP**, **Palser AL**, **Watson SJ**, **Lai IY-C**, **Gray ER**, **Grant P**, **Kanda RK**, **Leproust E**, **Kellam P**, **Breuer J**. 2011. Specific capture and whole-genome sequencing of viruses from clinical samples. PLoS ONE **6**:e27805.

2. **Pearson WR**, **Wood T**, **Zhang Z**, **Miller W**. 1997. Comparison of DNA sequences with protein sequences. Genomics **46**:24–36.

3. **Martin M**. 2011. Cutadapt removes adapter sequences from high-throughput sequencing reads. EMBnetjournal **17**:pp. 10–12.

4. **Parkhill J**, **Achtman M**, **James KD**, **Bentley SD**, **Churcher C**, **Klee SR**, **Morelli G**, **Basham D**, **Brown D**, **Chillingworth T**, **Davies RM**, **Davis P**, **Devlin K**, **Feltwell T**, **Hamlin N**, **Holroyd S**, **Jagels K**, **Leather S**, **Moule S**, **Mungall K**, **Quail MA**, **Rajandream MA**, **Rutherford KM**, **Simmonds M**, **Skelton J**, **Whitehead S**, **Spratt BG**, **Barrell BG**. 2000. Complete DNA sequence of a serogroup A strain of Neisseria meningitidis Z2491. Nature **404**:502–506.

5. **Benjamini Y**, **Hochberg Y**. 1995. Controlling the False Discovery Rate: A Practical and Powerful Approach to Multiple Testing. Journal of the Royal Statistical Society Series B (Methodological) **57**:289–300.

6. **Fagnocchi L**, **Bottini S**, **Golfieri G**, **Fantappiè L**, **Ferlicca F**, **Antunes A**, **Guadagnuolo S**, **Del Tordello E**, **Siena E**, **Serruto D**, **Scarlato V**, **Muzzi A**, **Delany I**. 2015. Global transcriptome analysis reveals small RNAs affecting Neisseria meningitidis bacteremia. PLoS ONE **10**:e0126325.

7. **Rusniok C**, **Vallenet D**, **Floquet S**, **Ewles H**, **Mouzé-Soulama C**, **Brown D**, **Lajus A**, **Buchrieser C**, **Médigue C**, **Glaser P**, **Pelicic V**. 2009. NeMeSys: a biological resource for narrowing the gap between sequence and function in the human pathogen Neisseria meningitidis. Genome Biol **10**:R110.

8. **Gil R**, **Silva FJ**, **Peretó J**, **Moya A**. 2004. Determination of the core of a minimal bacterial gene set. Microbiol Mol Biol Rev **68**:518–37– table of contents.
