# Supplementary figures and images for: Comprehensive Identification of Meningococcal Genes and Small Noncoding RNAs Required for Host Cell Colonization
Source: mBio. 2016 Aug 2;7(4):e01173-16. doi: 10.1128/mBio.01173-16 (PMC4981724; doi:10.1128/mBio.01173-16)

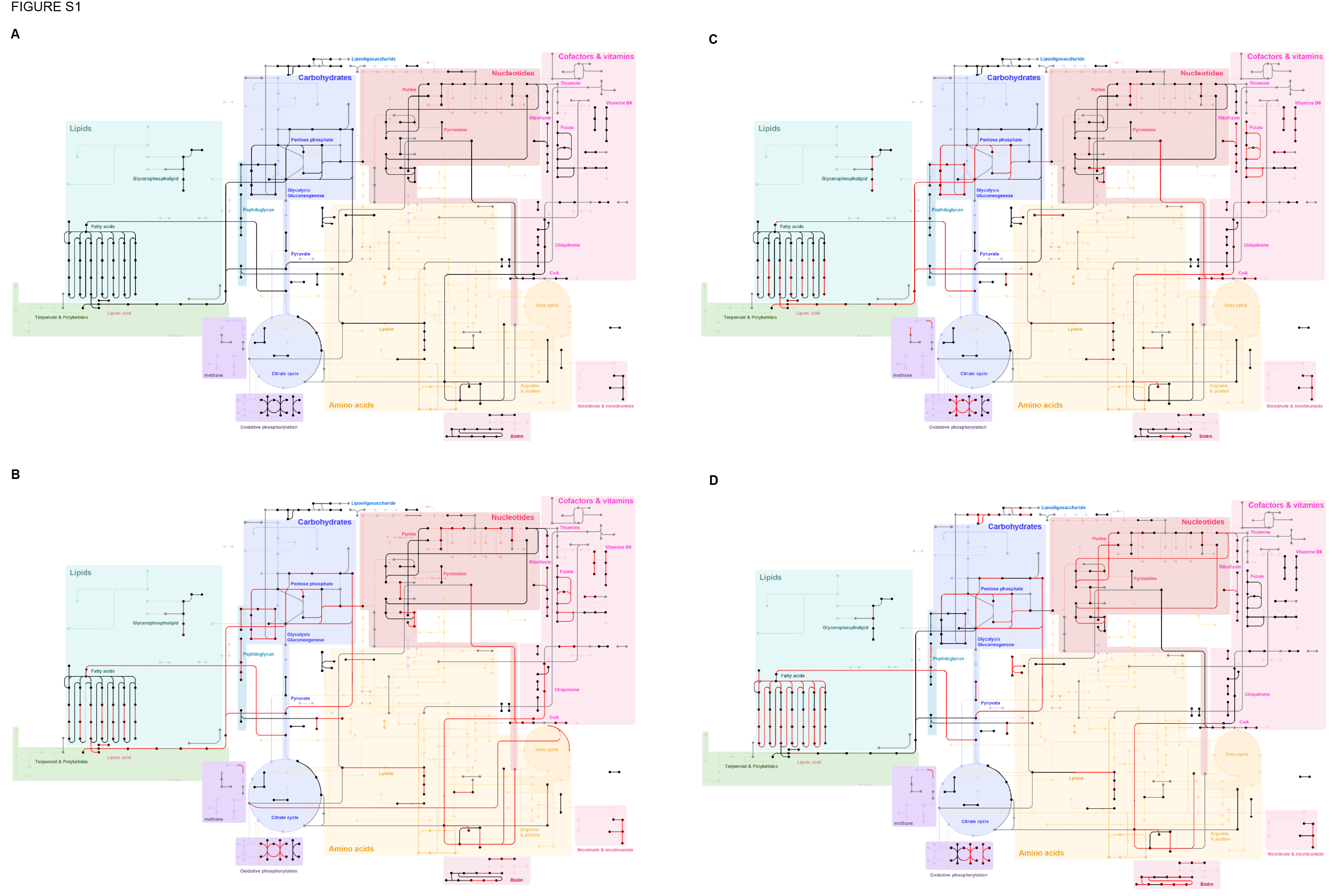

Supplement: Figure S1 — Metabolic overview of genes that contribute to N. meningitidis growth in GCB agar and CCM. Download [file mbo004162920sf1.tif]

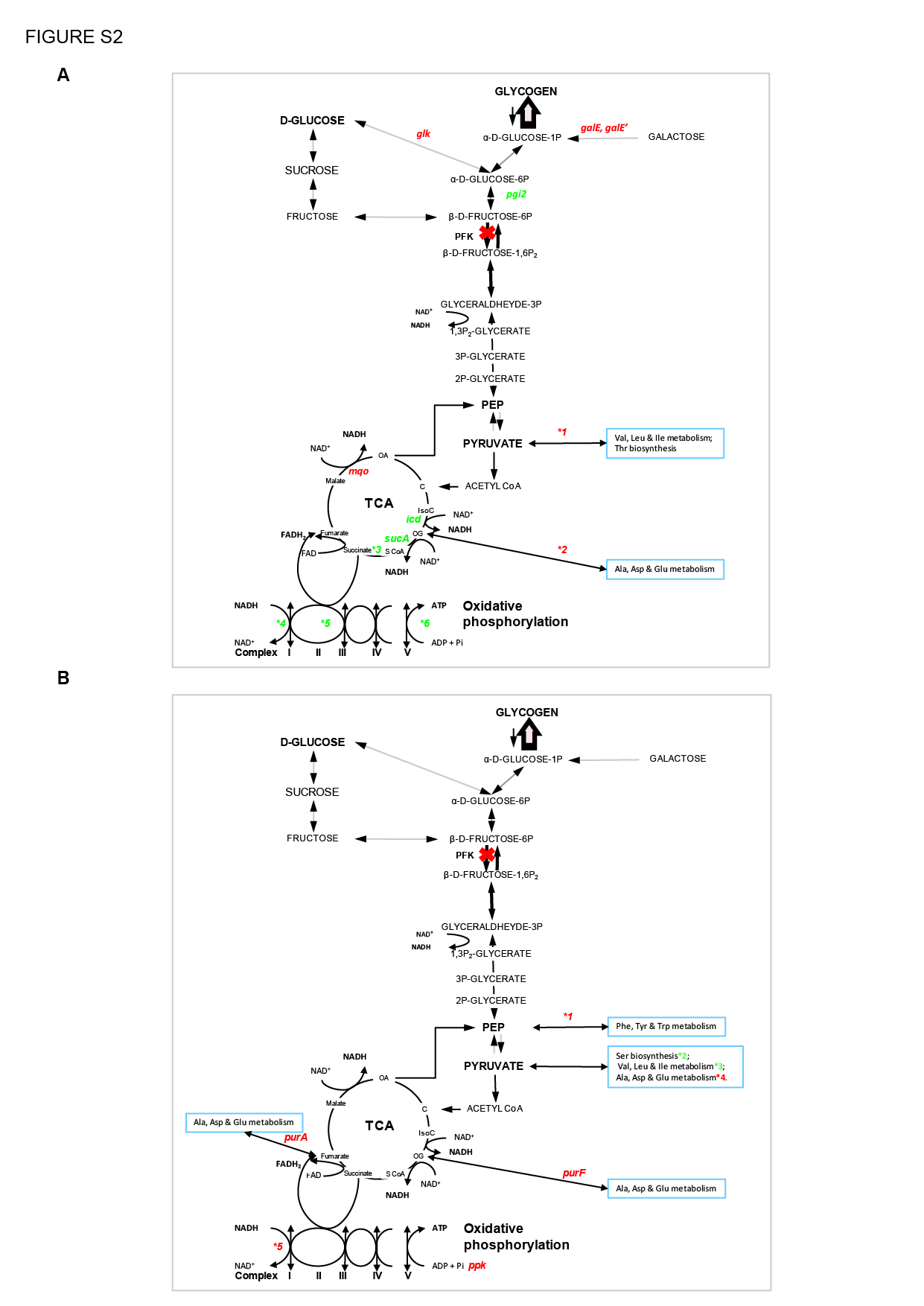

Supplement: Figure S2 — Schematic representation of the main metabolic pathways of conditional essential genes necessary for colonization of epithelial and endothelial cells. Download [file mbo004162920sf2.tif]
